# A Novel Role for UNC119 as an Enhancer of Synaptic Transmission

**DOI:** 10.3390/ijms24098106

**Published:** 2023-04-30

**Authors:** Katherine E. Fehlhaber, Anurima Majumder, Kimberly K. Boyd, Khris G. Griffis, Nikolai O. Artemyev, Gordon L. Fain, Alapakkam P. Sampath

**Affiliations:** 1 Department of Ophthalmology, Jules Stein Eye Institute, University of California, Los Angeles, CA 90095, USAgfain@ucla.edu (G.L.F.); 2 Molecular Physiology and Biophysics, University of Iowa, Iowa City, IA 52242, USAnikolai-artemyev@uiowa.edu (N.O.A.); 3 Ophthalmology and Visual Sciences, University of Iowa, Iowa City, IA 52242, USA

**Keywords:** bipolar cell, retina, synaptic transmission, adaptation, sensitivity, UNC119

## Abstract

Mammalian UNC119 is a ciliary trafficking chaperone highly expressed in the inner segment of retinal photoreceptors. Previous research has shown that UNC119 can bind to transducin, the synaptic ribbon protein RIBEYE, and the calcium-binding protein CaBP4, suggesting that UNC119 may have a role in synaptic transmission. We made patch-clamp recordings from retinal slices in mice with the *UNC119* gene deleted and showed that removal of even one gene of *UNC119* has no effect on the rod outer segment photocurrent, but acted on bipolar cells much like background light: it depolarized membrane potential, decreased sensitivity, accelerated response decay, and decreased the Hill coefficient of the response–intensity relationship. Similar effects were seen on rod bipolar-cell current and voltage responses, and after exposure to bright light to translocate transducin into the rod inner segment. These findings indicate that *UNC119* deletion reduces the steady-state glutamate release rate at rod synapses, though no change in the voltage dependence of the synaptic Ca current was detected. We conclude that UNC119, either by itself or together with transducin, can facilitate the release of glutamate at rod synapses, probably by some interaction with RIBEYE or other synaptic proteins rather than by binding to CaBP4 or calcium channels.

## 1. Introduction

Mammalian UNC119 (or MUNC119 or RG4) is a ciliary trafficking factor, which can facilitate the transport of myristolated protein cargo targeted by primary cilium [1,2,3]. Its UNC119a isoform is highly expressed in the inner segments of photoreceptors, where it is present at the level of about 1 molecule per 4 molecules of the heterotrimeric G-protein transducin [4]. UNC119 in photoreceptors is thought to facilitate the transport of transducin back into outer segments [5]. Transducin has been shown to diffuse from the rod outer segment to the inner segment in bright light (see for example [6,7]), and the return of transducin back to the outer segment in subsequent darkness is delayed in rods lacking UNC119 [8].

UNC119 is also present in the synaptic terminals of photoreceptors [4,9] and has been shown to interact with and bind to the protein RIBEYE [10]. RIBEYE is an essential component of the synaptic ribbon of photoreceptors [11,12], and photoreceptors without RIBEYE have no ribbons and greatly altered synaptic transmission [13]. These observations suggest that UNC119 may have a role in synaptic transmission in the retina.

To examine this possibility, we made patch-clamp recordings from rod photoreceptors and rod bipolar cells in slices of the retina from mice in which the gene for UNC119 has been deleted. We showed that synaptic transmission between the rods and bipolar cells persists but is altered, as if the rate of continuous release of glutamate at the rod synapse were reduced. Moreover, the facilitation of release by transducin after bright light exposure is largely ablated when UNC119 is absent. Our results suggest that UNC119 may be essential for maintaining the normal level of glutamate release at the photoreceptor ribbon synapse.

## 2. Results

### 2.1. Characterization of UNC119^−/−^ Retina

**Figure 1:**
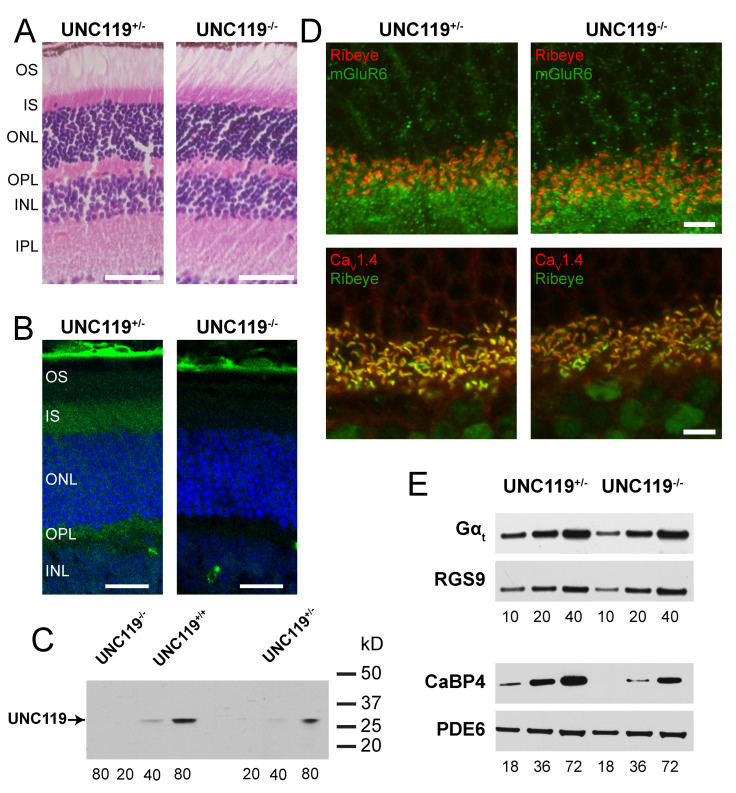
Characterization of *UNC119* knockout mouse retinas. (**A**) Retinal morphology of *UNC119^+/−^* (control) and *UNC119^−/−^* mice. INL, inner nuclear layer; IPL, inner plexiform layer; IS, rod inner segments; ONL, outer nuclear layer; OPL, outer plexiform layer; and OS, rod outer segments. (**B**) Localization of UNC119 in dark-adapted retinas. Retinas have been stained with a monoclonal antibody for UNC119 [14], followed by goat anti-rabbit AlexaFluor 546. (**C**) Western blot with antibody for UNC119. Numbers on right are molecular weights in kD determined from BSA and carbonic anhydrase standards. Numbers below lanes are μg soluble retinal protein. (**D**) Localization of rod-to-rod-bipolar-cell synaptic proteins Ribeye, mGluR6, and Ca_V_1.4 in *UNC119^+/−^* and *UNC119^−/−^* mice from immunofluorescence. (**E**) Immunoblot analysis of expression of various photoreceptor transduction proteins, such as Gα_t_, RGS9, CaBP4, and PDE6 in *UNC119^+/−^* and *UNC119^−/−^* mice. Numbers below lanes are μg soluble retinal protein. Magnification: 40×.

To study the role of UNC119 as a mediator of synaptic transmission, we utilized a mouse *UNC119^−/−^* model that lacked UNC119 (see Materials and Methods). Mice used for our recordings were 2–4 months of age, at which time *UNC119^−/−^* retinas were indistinguishable in morphology from *UNC119^+/−^* and WT retinas (see Figure 1A and reference [8]). In *UNC119^+/−^* retinas, UNC119 antibody labeled rods predominantly in the inner segment (IS) and at the rod synaptic terminal in the outer plexiform layer (OPL), as others have previously described [4,9]. This labeling is absent in *UNC119^−/−^* mice, both in retinal slices (Figure 1B) and in Western blots from whole retinas (Figure 1C, left); expression of UNC119 in *UNC119^+/−^* retinas is about half that in WT (Figure 1C, middle and right). Despite the absence of UNC119 in *UNC119^−/−^* retinas, the synapses between rod photoreceptors and rod bipolar cells appeared normal, since the distributions in immunofluorescence staining of the synaptic proteins RIBEYE, mGluR6, and Ca_V_1.4 were indistinguishable in *UNC119^−/−^* retinas and *UNC119^+/−^* retinas (Figure 1D). *UNC119^−/−^* retinas also expressed normal levels of the photoreceptor transduction proteins PDE6 and RGS9 (Figure 1E).

Previous studies have shown that UNC119 expression levels are reduced by about a factor of two in *Gnat1^−/−^* retinas lacking the gene for the alpha subunit of the G-protein transducin [4]. In *UNC119^−/−^* retinas, however, the expression of transducin alpha (Ga_t_) was similar to that in *UNC119^+/−^* retinas (Figure 1E), which is consistent with the much higher abundance of transducin than of UNC119 in rods [4]. There is also evidence that expression of UNC119 is diminished in retinas lacking CaBP4 [14], a calcium-binding protein expressed in photoreceptors and localized to synaptic terminals [15]. We found that, in *UNC119^−/−^* retinas, CaBP4 expression was reduced by about 1.7-fold (Figure 1E). 

**Figure 2:**
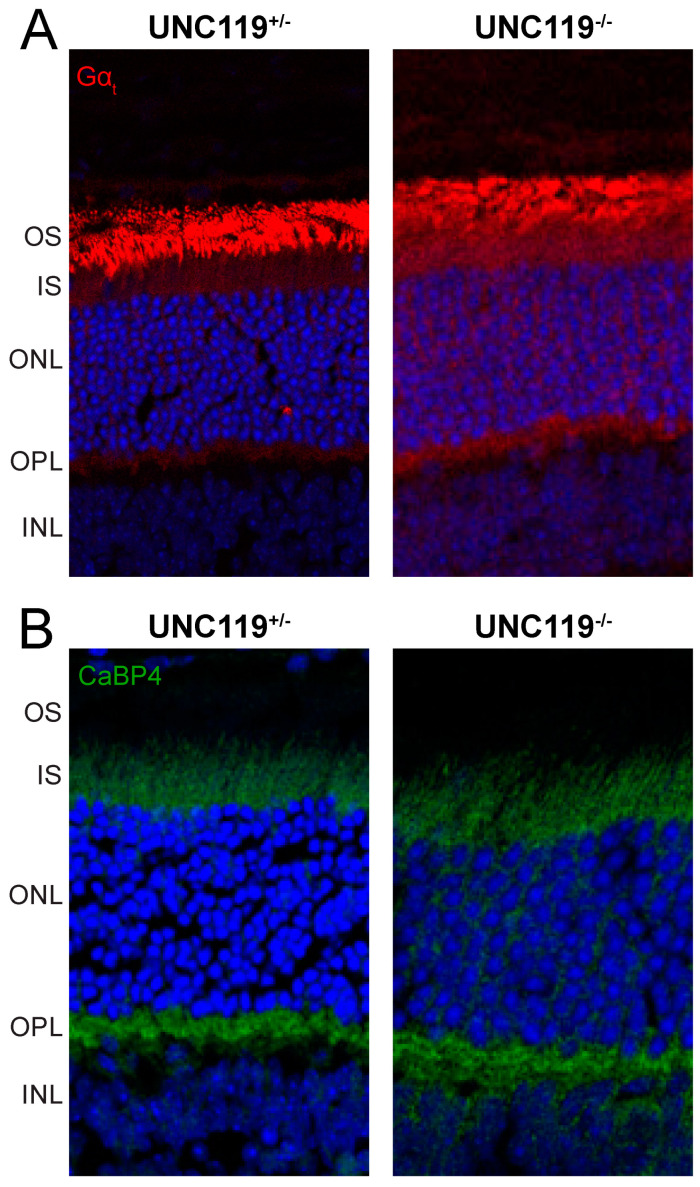
Localization of transducin (**A**) and CaBP4 (**B**) in dark-adapted retinas of *UNC119^+/−^* and *UNC119^−/−^* mice with immunofluorescence with anti-Gα_t_ and CaBP4 antibodies. INL, inner nuclear layer; IPL, inner plexiform layer; IS, rod inner segments; ONL, outer nuclear layer; OPL, outer plexiform layer; and OS, rod outer segments. Magnification: 40×.

Because of the role of UNC119 in assisting transducin return to outer segments [8], and the importance of CaBP4 in mediating synaptic transmission between rods and rod bipolar cells [15], we also examined possible changes in the distribution of these proteins in *UNC119^−/−^* retinas (Figure 2). In the dark-adapted *UNC119^+/−^* retina, the transducin alpha subunit, Gα_t_, is found almost exclusively in the outer segment (Figure 2A, left). Deletion of *UNC119* alters this distribution (Figure 2A, right), bringing more Gα_t_ into the inner segment and synaptic terminal, as was also observed by Zhang and colleagues [8]—see their Figure 6b. We found no significant change in the distribution of CaBP4 in the absence of UNC119 (Figure 2B).

### 2.2. Effect of UNC119 Deletion on Dark-Adapted Current Responses of Rods and Rod Bipolar Cells

**Figure 3:**
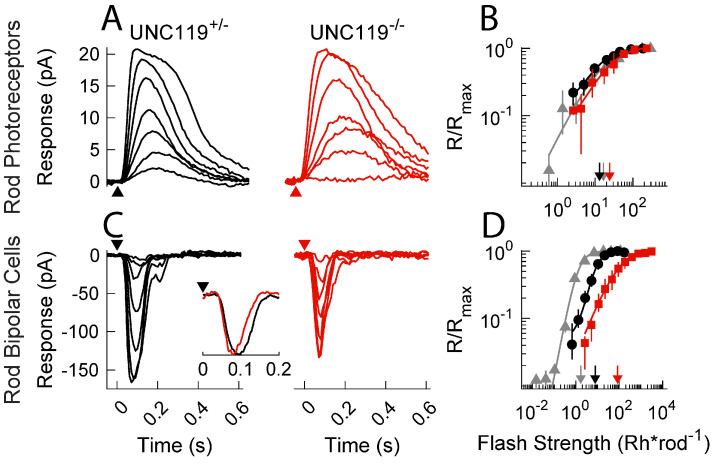
Photocurrent responses of rods and rod bipolar cells in the dark-adapted retina. (**A**) Representative flash response families from littermate control *UNC119^+/−^* (black) and *UNC119^−/−^* (red) rods in the dark-adapted retina. Timing of 20 ms flashes indicated by arrowheads. Intensities were 2.6, 5.2, 10, 21, 42, 83, and 170 Rh* rod^−1^ for *UNC119^+/−^* rods and 3.9, 7.8, 16, 31, 62, 120, and 250 Rh* rod^−1^ for *UNC119^−/−^* rods. (**B**) Response–intensity relationships for dark-adapted rods from WT (gray), *UNC119^+/−^* (black) and *UNC119^−/−^* (red) mice. Normalized response was plotted against calculated flash intensity in Rh* rod^−1^. Sensitivity was estimated from the half-maximal flash strength of the Hill curve (Materials and Methods, Eqn. 1) as indicated by downward arrows. (**C**) Representative flash response families from *UNC119^+/−^* and *UNC119^−/−^* rod bipolar cells. Intensities of *UNC119^+/−^* were 0.66, 1.3, 2.7, 5.3, 11, 21, and 42 Rh* rod^−1^ and for *UNC119^−/−^* were 12, 23, 47, 93, 190, 370, and 750 Rh* rod^−1^. Inset: representative response waveforms to dim flashes for *UNC119^+/−^* (black) and *UNC119^−/−^* (red) rod bipolar cells. (**D**) Response–intensity relationships as in (**B**) but from bipolar cells of WT (gray), *UNC119^+/−^* (black) and *UNC119^−/−^* (red) mice with half-maximal flash strength of the Hill curve indicated by downward arrows.

To analyze the role of UNC119 in modulating synaptic transmission, we performed single-cell, patch-clamp recordings from dark-adapted retinal slices as in previous studies [16,17]. *UNC119^−/−^* rod photocurrents had similar response characteristics to both WT and *UNC119^+/−^* littermates (Figure 3A). Peak response amplitudes averaged 26 pA (13, 36; n = 4) for *UNC119^−/−^* rods, 30 pA (19, 61; n = 21) for *UNC119^+/−^* littermates, and 24 pA (22, 25; n = 10) for WT. Note that here and elsewhere in the manuscript, the value of the mean is followed by the confidence interval (see Materials and Methods, 4.7, Statistics). None of these differences was significant. The sensitivities of rods in the retinas of these two strains were also not significantly different (see Figure 3B), with an *I*_1/2_ of 19 Rh* rod^−1^ (12, 27; n = 4) for *UNC119^−/−^* rods, 11 Rh* rod^−1^ (9.1, 14; n = 6) for *UNC119^+/−^* littermates, and 17 Rh* rod^−1^ (11, 18; n = 10) for WT. These results contrast with those from rods lacking the calcium-binding protein CaBP4 [15], whose responses declined more rapidly than those of control rods and were less sensitive.

We saw a more dramatic change in response properties of rod bipolar cells from *UNC119^−/−^* and control retinas (Figure 3C). Mean peak response amplitudes were similar: −100 pA (−130, −77; n = 6) for *UNC119^−/−^* retinas, −110 pA (−150, −77; n = 15) for *UNC119^+/−^* littermate, and −180 pA (−250, −140; n = 46) for WT. There were, however, three significant differences. (1) Responses for *UNC119^−/−^* and *UNC119^−/−^* rod bipolar cells were less sensitive than those in control retinas (Figure 3D). The light intensities required to produce a half-maximal response (*I*_1/2_) were 73 Rh* rod^−1^ (41, 100; n = 21) for *UNC119^−/−^* rod bipolar cells, 9.9 Rh* rod^−1^ (7.7, 13; n = 15) for *UNC119^+/−^* littermate rod bipolar cells (*p* = 0.0002), and 1.3 Rh* rod^−1^ (1.2, 1.4; n = 46) for WT rod bipolar cells (*p* = 0.017 for *UNC119^+/−^*, 0.0001 for *UNC119^−/−^*), indicating an approximate 7 to 8-fold decrease in sensitivity, first between WT and *UNC119^+/−^* rod bipolar cells and then again between *UNC119^+/−^* and *UNC119^−/−^* rod bipolar cells. (2) The shape of the response–intensity curve was altered. Data from cells of each strain could be fitted with the Hill equation (Eqn. 1 of Materials and Methods), but the value of the Hill coefficient *n* was 0.62 (0.46, 0.85; n = 21) for *UNC119^−/−^* rod bipolar cells, 1.4 (1.0, 2.0; n = 15) for rod bipolar cells in *UNC119^+/−^* littermates (*p* = 0.0001), and 1.7 (1.6, 1.9; n = 46) for WT—significantly different from *UNC119^−/−^* but not from *UNC119^+/−^*—see also [16,17]. (3) Photoresponses decayed more rapidly. This difference can be seen by comparing the waveforms of the black and red responses in Figure 3C. Measurements of the time to peak for half-saturating responses gave 78 ms (75, 82, n = 15) for *UNC119^−/−^* bipolar cells but 97 ms (84, 110, n = 21) for *UNC119^+/−^* littermates (*p* = 0.0192). In addition, we show in the inset to Figure 3C normalized response waveforms from two representative cells for small-amplitude responses. The accelerated decay of the response of the *UNC119^−/−^* bipolar cell (red) is clear. 

The comparison to mice lacking the CaBP4 protein is again instructive [15]. Responses of *CaBP4^−/−^* rod bipolar cells are smaller and less sensitive than those in control retinas, and the slope of the response–intensity curve is again more gradual. The decay of the response waveform was, however, slower than in control retinas, even though responses of *CaBP4^−/−^* rods decayed more rapidly. Thus, the effects on response decay for rod bipolar cells were opposite in *CaBP4^−/−^* to those produced in *UNC119^−/−^* retina.

### 2.3. Effect of Transducin Translocation

Majumder and colleagues [16] exposed the retina to light bright enough to bleach a substantial fraction of the photopigment to cause the translocation of much of the transducin from the rod outer segment to the inner segment. After waiting to allow rods to recover some sensitivity, but without significant return of transducin back into the outer segment, they recorded light responses from both rods and rod bipolar cells. They showed that the rods were desensitized, presumably from activation of the phototransduction cascade by photopigment that had been bleached but not yet regenerated [18,19], but that bipolar cells were paradoxically almost unaffected by the bleaching exposure. They hypothesized that the translocation of transducin into the rod inner segment could in some way facilitate synaptic transmission to the rod bipolar cells.

UNC119 is known to interact with transducin [8,20], and it seemed possible to us that deletion of UNC119 might prevent at least part of the sensitizing effect of transducin on synaptic transmission. To test this notion, we exposed mice to the same protocol used by Majumder and colleagues [16] to bleach photopigment and cause transducin translocation; we then waited 30 min for about 65% of the bleached pigment to regenerate but before a substantial fraction of transducin could be returned to the outer segment (see Materials and Methods). 

**Figure 4:**
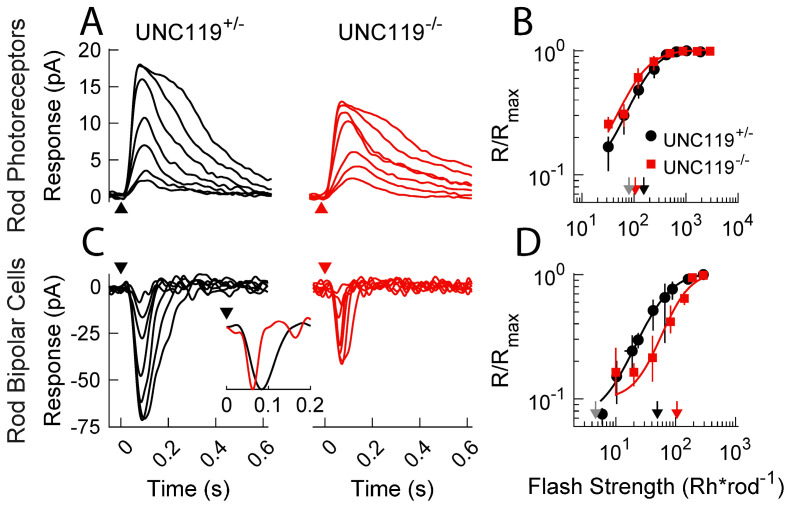
Current responses of rods and rod bipolar cells after exposure to bright light. Mice were exposed to bright bleaching light to translocate transducin to the rod inner segments and were then placed in darkness for 30 min to allow recovery of about 65% of the visual pigment. (**A**) Representative flash response families from littermate *UNC119^+/−^* (black) and *UNC119^−/−^* (red) rods in the dark-adapted retina. Timing of 20 ms flashes indicated by arrowheads. Intensities were 32, 64, 130, 260, 420, 600, 900, and 1900 Rh* rod^−1^ for *UNC119^+/−^*; and 32, 64, 130, 260, 420, 600, 900, 1900, and 2900 Rh* rod^−1^ for *UNC119^−/−^*. (**B**) Response–intensity relationships for dark-adapted rods from *UNC119^+/−^* (black) and *UNC119^−/−^* (red) mice, plotted as in Figure 3B. Downward arrows give best-fitting values of *I*_1/2_ from Eqn. (1). Included for comparison is the value of *I*_1/2_ for WT rods (gray) provided in Majumder et al. [16]. (**C**) Representative flash response families from *UNC119^+/−^* and *UNC119^−/−^* rod bipolar cells. Flash intensities for *UNC119^+/−^* were 5.5, 11, 22, 44, 72, 100, and 150 Rh* rod^−1^ for *UNC119^+/−^* and 10, 20, 41, 82, 140, 190, and 290 Rh* rod^−1^ for *UNC119^−/−^*. Inset: representative response waveforms for *UNC119^+/−^* (black) and *UNC119^−/−^* (red) rod bipolar cells to dim flashes. (**D**) Response–intensity relationships for light-adapted bipolar cells as in (**C**) from *UNC119^+/−^* (black) and *UNC119^−/−^* (red) mice. Downward arrows give best-fitting values of *I*_1/2_ for rod bipolar cells in *UNC119^+/−^* (black) and *UNC119^−/−^* (red) mice, with comparison to the value in WT rods (gray) from Majumder et al. [16].

The results of these experiments are shown in Figure 4. After the bleaching exposure, the control and *UNC119^−/−^* rods displayed similar waveforms (Figure 4A) and sensitivities (Figure 4B) to one another and to WT rods, which, under these conditions, had an *I*_1/2_ of 80 ± 3.0 Rh* rod^−1^, see [16]. The *I*_1/2_ of the WT rods is indicated in Figure 4B by the gray, downward pointing arrow adjacent to the arrows for the *UNC119* mutants. All these values of *I*_1/2_ are much larger than for rods in dark-adapted retina (see Figure 3B). 

In WT rod bipolar cells light-adapted with an identical protocol as in the experiments of Majumder et al. [16], the value of the intensity producing a half-maximal response (*I*_1/2_) was 4.6 ± 0.3 Rh* rod^−1^ (n = 7), indicated by the gray arrow in Figure 4D. Comparison to Figure 3D shows that the light-adaptation protocol had almost no effect on the WT rod bipolar-cell sensitivity, despite the large effect on the sensitivity of the rods. In mice lacking one or both UNC119 genes, on the other hand, the bipolar cells were much less sensitive. The light intensity required to produce a half-maximal response was 105 Rh* rod^−1^ (94, 117; n = 7) for *UNC119^−/−^* rod bipolar cells and 50 Rh* rod^−1^ (40, 60; n = 7) for bipolar cells in *UNC119^+/−^* littermates (*p* = 0.0138, see Figure 4D). Responses of *UNC119^−/−^* bipolar cells had a significantly shorter time to peak than *UNC119^+/−^* bipolar cells (*p* = 0.027), much as in dark-adapted retina (Figure 3C). This difference is clearly evident in the inset to Figure 4C, which shows representative response waveforms for *UNC119^+/−^* (black) and *UNC119^−/−^* (red) rod bipolar cells to dim flashes. 

These findings indicate that removal of even a single copy of the *UNC119* gene eliminates much of the bipolar-cell sensitization thought to be produced by migration of transducin into the inner segment after bright light exposure. The sensitization effect of transducin seems therefore to require the presence of UNC119.

### 2.4. Measurement of Voltage Responses

The experiments we have described so far indicate that UNC119 can facilitate synaptic transmission between the rod and rod bipolar cell either in darkness (Figure 3) or after exposure to bright illumination (Figure 4). In the dark-adapted retina, deletion of UNC119 has many of the same effects on rod bipolar cells as exposure to background light [21]: the response was less sensitive, the Hill coefficient of the response–intensity curve decreased, and response decay was accelerated. These results suggest that UNC119 may achieve its effect by decreasing the steady-state release of glutamate from the rod synaptic terminal, whether in the dark or after strong illumination.

**Figure 5:**
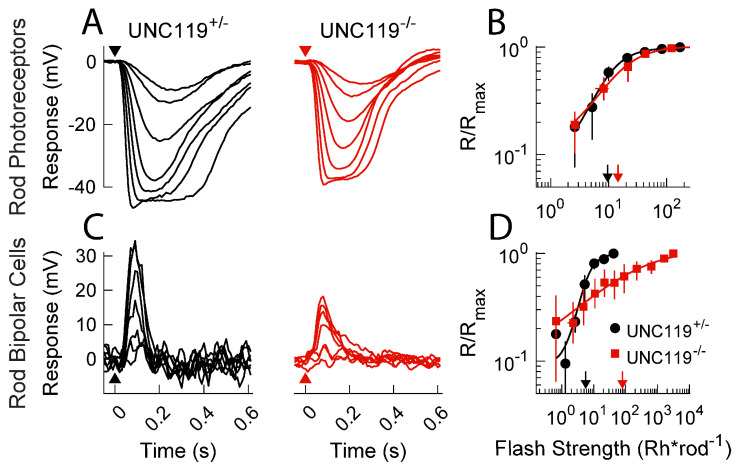
Photovoltage responses of rods and rod bipolar cells in the dark-adapted retina. (**A**) Representative flash response families from heterozygous littermate controls (*UNC119^+/−^*) and UNC119 knockout (*UNC119^−/−^*) rods in the dark-adapted retina. Timing of 20 ms flashes indicated by arrowheads. Intensities were 2.6, 5.2, 10, 21, 42, 83, and 170 Rh* rod^−1^ for both *UNC119^+/−^* and *UNC119^−/−^*. (**B**) Response–intensity relationships for dark-adapted rods from *UNC119^+/−^* (black) and *UNC119^−/−^* (red) mice. Sensitivity (*I*_1/2_) is indicated by downward arrows. (**C**) Representative flash response families from *UNC119^+/−^* and *UNC119^−/−^* rod bipolar cells. Intensities of 20 ms flashes were 0.66, 1.3, 2.7, 5.3, 11, 21, and 42 Rh* rod^−1^ for *UNC119^+/−^* (black) and 49, 97, 190, 390, 780, 1600, and 3100 Rh* rod^−1^ for *UNC119^−/−^* rod bipolar cells (**D**) Response–intensity relationships as in (**B**) for dark-adapted rod bipolar cells from *UNC119^+/−^* (black) and *UNC119^−/−^* (red) mice.

To test this hypothesis, we recorded photovoltage responses from rods and rod bipolar cells in a current-clamp mode (Figure 5). Photovoltage responses of *UNC119^−/−^* rods and rod bipolar cells were similar to photocurrent responses (Figure 3). Deletion of *UNC119* had no effect on rod photovoltages (Figure 5A,B), but *UNC119^−/−^* rod bipolar cells were significantly less sensitive, with an *I*_1/2_ of 110 Rh* rod^−1^ (94, 120; n = 12) compared to 50 Rh* rod^−1^ (40, 60; n = 5) for *UNC119^+/−^* littermates (*p* = 0.0043). Moreover, the Hill coefficient *n* was also altered (Figure 5D): it was 0.4 (0.4, 0.4; n = 12), for *UNC119^−/−^*, but 2 (1.9, 2; n = 5) for *UNC119^+/−^* littermates (*p* = 0.0003), indicating that response–intensity curves were far less steep, as observed also for photocurrent responses (Figure 3D). 

**Figure 6:**
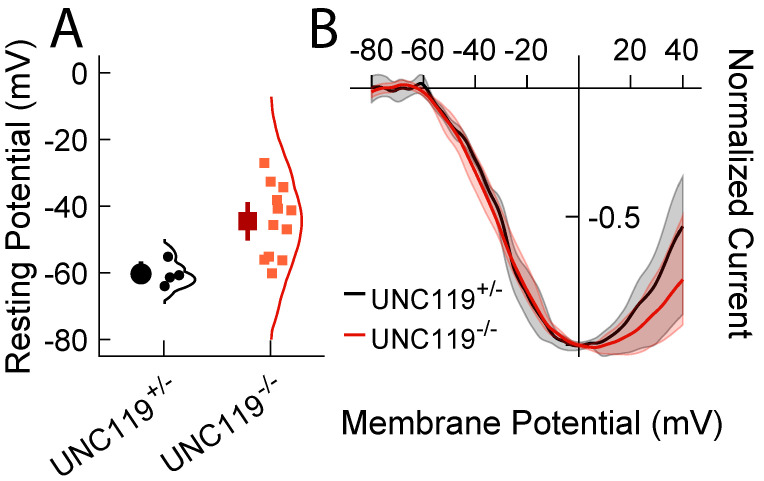
(**A**) Resting membrane potentials of rod bipolar cells from *UNC119^+/−^* (**left**) and *UNC119^−/−^* (**right**) mice. Large symbols give mean with 95% confidence intervals. Smaller symbols are individual measurements clustered as shown. (**B**) Ca^2+^ currents recorded from *UNC119^+/−^* (black) and *UNC119^−/−^* (red) rods with voltage ramps (see Materials and Methods). Shaded regions are 95% confidence limits.

In addition to these effects, and perhaps of greatest significance, the resting membrane potential was more depolarized in *UNC119^−/−^* rod bipolar cells (Figure 6A), having a mean value of −45 mV (−50, −39; n = 12) in comparison to −60 mV (−63, −57; n = 5) for *UNC119^+/−^* littermates. This difference was highly significant (*p* = 0.0014). These values should be compared with measurements of resting potential in WT retinas of −61 ± 2.5 of Arman and Sampath [22], nearly the same value as we obtained for the *UNC119^+/−^* littermates and about 15 mV more negative than for the *UNC119^−/−^* bipolar cells. Since the continuous release of glutamate from the rod synaptic terminal is known to produce a persistent closure of cationic TRPM1 channels in the bipolar cell dendrites, keeping the bipolar-cell membrane potential relatively hyperpolarized [23], a decrease in glutamate release would produce a net membrane-potential depolarization similar to that observed in the *UNC119^−/−^* retina.

### 2.5. Lack of Effect of Deletion of UNC119 on Voltage Dependence of Calcium Current

Since UNC119 is known to bind to the protein RIBEYE at the rod synaptic terminal [10], and since our experiments indicate that UNC119 may modulate the rate of release of glutamate, we wondered whether UNC119 affects the voltage dependence of the rod synaptic Ca_V_1.4 calcium channels. Deletion of CaBP4 is known to reduce glutamate release by shifting the activation curve of the Ca current to more depolarized potentials [15]. Since UNC119 is known to bind to CaBP4 [14], we thought it possible that UNC119 could affect the voltage dependence of the Ca channels in a similar fashion.

We therefore measured the voltage dependence of the Ca current with voltage ramps. Rod membrane potential was continuously increased from −80 to +40 mV during 1000 ms, after blocking other voltage-dependent currents as previously described—see Materials and Methods and [16]. Residual linear currents were then subtracted from the record, and the remaining current was plotted as a function of voltage (Figure 6B). We could detect no difference between the voltage dependence of the currents of *UNC119^−/−^* rods and *UNC119^+/−^* littermates. We conclude that UNC119 affects glutamate release by another mechanism, perhaps via a direct interaction with RIBEYE or other proteins of the synaptic ribbon or release sites.

## 3. Discussion

We used patch-clamp recordings from mouse retinal slices to examine the effect of deletion of the gene for UNC119 on synaptic transmission between mouse rods and rod bipolar cells. We showed that removal of UNC119 had no effect on rod light responses but acted on bipolar-cells much like background light [21]: it depolarized the rod bipolar cell membrane potential, decreased sensitivity, accelerated response decay, and decreased the Hill coefficient of the response–intensity relationship (Figure 3, Figure 5 and Figure 6A). Some of these effects were seen at least in part even in the *UNC119* heterozygotes. Similar effects were observed after exposure of bright light to produce translocation of transducin into the rod inner segment (Figure 4), indicating that UNC119 may also modulate the facilitation of synaptic transmission by transducin [16]. We conclude that UNC119 can potentiate transmitter release at the rod synapse in the presence of transducin after transducin translocation, but also in dark-adapted retinas with minimal transducin in the rod inner segment. Although the mechanism of UNC119 modulation is still unclear, we could detect no effect of UNC119 deletion on the voltage dependence of the rod Ca_V_1.4 calcium channel, and we think it unlikely that UNC119 works by binding to CaBP4 or to the Ca_V_1.4 channels. We think it more probable that UNC119 achieves its effect by binding to RIBEYE [10] or other proteins mediating glutamate release at the rod synaptic ribbon [24].

### 3.1. Modulation of Synaptic Transmission by Transducin

Previous experiments have shown that translocation of transducin from the rod outer segment to the inner segment in bright light can alter synaptic transmission from the rod to the rod bipolar cell [16]. When the retina is exposed to bright light and then placed in darkness to allow some regeneration of rhodopsin, rods are desensitized from activation of the phototransduction cascade by the fraction of bleached pigment remaining unregenerated [18,19]. Rod bipolar cells, in contrast, seem nearly unaffected by the bleaching exposure. This earlier work also showed that when translocation of transducin is prevented, this effect disappears, and the bleaching exposure desensitizes rods and rod bipolar cells nearly equally. These experiments show that accumulation of transducin in the rod inner segment can facilitate transmitter release from the rods to the rod bipolar cells. 

Our experiments demonstrate that this facilitation by transducin is altered by deletion of the gene for UNC119 (Figure 4). Photoreceptors after bright illumination are desensitized to the same extent with and without UNC119 (Figure 4A), but bipolar cells are much more desensitized in animals lacking UNC119 (Figure 4B). One possible interpretation of this result is that transducin in the inner segment facilitates synaptic transmission by binding to UNC119 [8,20,25]. Our results also show, however, that UNC119 can facilitate transmission in the dark-adapted retina (Figure 3 and Figure 5), when there is little transducin in the inner segment (see for example Figure 2A). It would therefore seem that UNC119 can have an effect by itself without transducin, but that this effect is potentiated when transducin is present after bright light exposure. Besides UNC119, other transducin partners in rod photoreceptors, such as GPSM2 and Frmpd1, may also modulate signal transmission to the rod bipolar cell after transducin translocation [26].

### 3.2. Role of CaBP4 and Calcium Channels

The effect of *UNC119* deletion resembles in many respects the effect of deletion of the gene for CaBP4, a calcium-binding protein expressed in photoreceptors and localized to synaptic terminals [15]. Deletion of CaBP4 decreases the sensitivity and amplitude of bipolar cell responses, apparently by altering the voltage dependence of the synaptic Ca channels and reducing the rate of release of glutamate from the rod synaptic terminal. Since deletion of UNC119 decreases the expression of CaBP4 (Figure 1E), it would seem possible that the effect of UNC119 deletion results from the reduction of CaBP4 expression. This interpretation seems to us unlikely for three reasons. First, the decrease in CaBP4 expression produced by *UNC119* deletion is modest in comparison to deletion of the *CaBP4* gene. It would seem surprising if such a small effect were responsible for the large decrease in sensitivity produced by *UNC119* deletion. Second, the effects on photoresponse decay for rods and rod bipolar cells in the *CaBP4^−/−^* retina were opposite to those produced in *UNC119^−/−^*. Photoreceptor responses may decay somewhat more slowly in *UNC119^−/−^* (Figure 3A) but more rapidly in *CaBP4^−/−^* [15]; rod bipolar-cell responses, on the other hand, decayed more rapidly in *UNC119^−/−^* (Figure 3B, inset) but more slowly in *CaBP4^−/−^* [15]. Third, and perhaps of greatest importance, deletion of the gene for CaBP4 alters the voltage dependence of the rod Ca channels, shifting it to more hyperpolarized potentials [15]. This effect is likely to be responsible for the decrease in glutamate release in *CaBP4^−/−^* retina. In *UNC119^−/−^* rods, however, we could detect no change in the voltage dependence of the Ca current (Figure 6B).

### 3.3. Mechanism of Action of UNC119

UNC119 has been shown to be a ciliary trafficking chaperone associated with primary cilium, which can bind to myristolated proteins, including the alpha subunit of transducin, and promote dissociation of Gα_t_-GTP from the beta and gamma subunits [2,8,20]. UNC119 can also interact with ARL3 to release ciliary cargo [2,3]. In this way, UNC119 is thought to contribute to the transport of transducin from the inner segment to the outer segment [2,8]. UNC119 is present in the rod synaptic terminals and can bind to RIBEYE at the ribbon [9,10], where ARL3 is also localized [24]. These findings, together with our observations, suggest that UNC119 may have some specific role at the synapse in facilitating the transport of synaptic proteins or vesicles. At present, however, too little is known about the molecular mechanisms underlying vesicle movement and release at the rod ribbon synapse to postulate a specific mechanism of action of UNC119, which remains a subject of future investigation.

## 4. Materials and Methods

### 4.1. UNC119Knockout Mice

All experiments involving mice were approved by the Institutional Animal Care and Use Committees at the University of Iowa and University of California, Los Angeles. The wild-type (WT) mice for Figure 3 were C57BL/6J purchased from The Jackson Laboratory (Bar Harbor, Maine, USA). Heterozygote *UNC119a* mice were purchased from UC Davis KOMP (Davis, CA, USA) and were bred to homozygous knockout *UNC119a*. For genotyping, mouse genomic DNA was PCR-amplified with the following primers: forward primer 1: ATCAGCCATATCACTCTGTAGAGG; reverse primer 2: TGGCCGCACCCA AGAACAGAA AAGG; forward primer 3: ACTGAAACTGAAGTGACAATGGGCG; and reverse primer 4: ATCCTCTCAACGGTGTACCAATCCC. PCR with primers 1 and 2 yields a 600bp band when the KO allele is present, whereas the product is 250bp with primers 3 and 4 in the presence of the WT allele. Mice were kept under a 12:12 h light/dark cycle in approved cages and supplied with ample food and water. Males and females were used in equal numbers. Mice employed for physiological recordings were between 2 and 4 months of age, before the onset of significant rod degeneration [27].

### 4.2. Immunofluorescence 

The procedures used for immunoblotting, immunofluorescence, retina morphology analyses, and rhodopsin regeneration assays are well established and have been previously described [16]. For immunofluorescence, eyeballs were enucleated from dark-adapted mice that were euthanized by CO_2_ asphyxiation. Corneas were pierced with a 21-gauge needle and fixed and embedded as previously [16]. Sections were first incubated in 0.1% Triton X-100 in PBS for 30 min, which was followed by a 1 h incubation with primary antibodies and subsequent staining with either goat anti-rabbit AlexaFluor 546, donkey anti-sheep AlexaFluor 488, or goat anti-mouse AlexaFluor 488 secondary antibodies (1:1000; Molecular Probes, Thermo Fisher Scientific Inc., Waltham, MA, USA) for 1 h. All sections were subsequently counterstained with TO-PRO3 nuclear stain. The stained sections were visualized with a Zeiss LSM 510 confocal microscope (Carl Zeiss, Stuttgart, Germany). The following primary antibodies were used: rabbit anti rod Gα_t1_ antibody K-20 (sc 389, 1:1000), and mouse RIBEYE (1: 500–1:1000 #612044, BD bioscience). A monoclonal antibody against UNC119a [14] and rabbit anti-CaBP4 [15] were kind gifts from Dr. Francoise Haeseleer (University of Washington), the sheep anti mGluR6 [28] was a kind gift from K. Martemyanov (Scripps Biomedical Research), and the rabbit Ca_V_1.4 antibody [29] was custom made in Dr. Amy Lee’s lab (University of Iowa, Iowa City, IA, USA). 

### 4.3. Immunoblotting

Total mouse retinal homogenates were obtained by solubilization of two retinas in 100 μL 10% SDS–Na with brief sonication. Protein concentrations were determined with the DC Protein Assay (Bio-Rad, Hercules, CA, USA). Samples of retinal homogenates were analyzed by immunoblotting with the following primary antibodies: anti-UNC119a and anti-CaBP4 (from Haeseleer lab), antirod Gα_t1_ (K-20) (Santa Cruz Biotechnology, Dallas, TX, USA), anti-phosphodiesterase-6 (PDE6) antibodies (Cytosignal, Irvine, CA, USA), and anti- regulatory of G protein signaling 9 (RGS9) antibodies (Elmira Biologicals, Iowa City, IA, USA). 

### 4.4. Retinal Morphology

Mice were euthanized by CO_2_ asphyxiation. Enucleated mouse eyeballs were fixed in 4% paraformaldehyde in PBS for 1 h at 25 °C, embedded in paraffin, sectioned (Leica RM2135, Wetzlar, Germany), stained with hematoxylin and eosin (H & E), and examined in an Olympus BX51 microscope (Shinjuku, Tokyo, Japan) under 40× magnification.

### 4.5. Physiological Recording from Rods and Rod Bipolar Cells

Currents of rods and rod bipolar cells were measured with patch electrodes and methods described previously [16,30,31,32]. Retinal tissues were superfused with Ames’ medium equilibrated with 5% CO_2_/ 95% O_2_ and maintained at 35 to 37 °C. The pipette internal solution consisted of 125 mM K-Aspartate, 10 mM KCl, 10 mM HEPES, 5 mM N-methyl glucamine-N-(2-hydroxyethyl)ethylenediamine-N,N,N-triacetic acid, 0.5 mM CaCl_2_, 1 mM ATP-Mg, and 0.2 GTP-Mg. The pH was adjusted to 7.3 with N-methyl-glucamine hydroxide. 

Light-evoked responses were generated either by delivering 10-ms flashes from a blue light-emitting diode (λ_max_ ~ 470 nm) of varying intensity, or by delivering flashes of variable duration ranging from 0.1 to 6.4 ms from a bluish green LED (λ_max_ ~ 500 nm). Records were sampled at 1 kHz and low pass-filtered at 50 Hz. Stimulus–response relations were evaluated from stimulus intensities in R* rod^−1^ and normalized response amplitudes. Relations were fit in the program Igor Pro (WaveMetrics, Portland, OR, USA) with a Hill model with 4 free parameters,

(1) $$\frac{R}{R_{max}}=R_{0}+\frac{(R_{s}-R_{0})\Phi^{n}}{\Phi^{n}+I_{1/2}^{n}}$$

where *R* is the peak amplitude of the response, *R_max_* is the maximum value of *R*, *Φ* is the strength or intensity of the flash, *R*_0_ is the initial response offset (typically near 0), *R_s_* is the maximal response (typically 1), *I*_1/2_ is the intensity producing a half-maximal response, and *n* is the Hill exponent which represents the nonlinearity coefficient of the relationship. All intensities were measured in photons µm^−2^ with a calibrated photodiode (Gamma Scientific, San Diego, CA, USA) through a photodiode amplifier (PDA200C; Thorlabs, Newton, NJ, USA) and converted into activated rhodopsins per rod, Rh* rod^−1^, with an effective collecting area of 0.3 µm^2^.

The voltage sensitivity of Ca^2+^ currents in rods was measured with patch-clamp electrodes from the rod inner segment in retinal slices. The rod membrane potential was continuously ramped from −80 to +40 mV in 1000 ms; a pipette internal solution blocked other cationic currents as previously described [16]. The internal solution consisted of 110 mM Cs-methanesulfonate, 12 mM tetramethylammonium-Cl, 10 mM Hepes, 10 mM EGTA, 2 mM QX-314, 11 mM ATP-Mg, 0.5 mM GTP·Tris, and 0.5 mM MgCl_2_. The pH was adjusted to 7.3 with N-methyl-glucamine hydroxide. Residual linear currents were subtracted, leaving voltage-sensitive currents which control experiments showed could be blocked completely by the Ca_V_1.4-channel blocker isradipine [33].

### 4.6. Light-induced Transducin Translocation

In some experiments, mice were exposed to light bright enough to cause transducin translocation as previously described [16]. Rhodopsin concentration was determined from absorption spectra before and after this translocation protocol by assuming an extinction coefficient at 500 nm of 42,000 M^−1^ cm^−1^. Visual pigment was regenerated up to about 65% of its dark-adapted level under these conditions [16]. Recordings were always terminated within 1 h after the translocation protocol had been given, in order to ensure that transducin remained substantially within the rod inner segment during the recordings [6].

### 4.7. Statistics

All uncertainties were calculated by Monte Carlo simulations (bootstrap) with 10,000 simulations, except for confidence regions for which 2000 simulations were used in the interest of computational time. To increase accuracy and mitigate errors that arise when data do not satisfy Gaussian assumptions, confidence intervals were estimated by the BCa method [34,35] and are given after the means in the text. In cases where fitting procedures were used, data were binned by logarithmic-spaced intervals, and fits were performed with a total least-squares method (also known as orthogonal regression) by the Total Least Squares Approach to Modeling Toolbox for MATLAB [36]. The fitting procedure was bootstrapped by resampling from the residuals of individual cells [37,38]. The data were resampled, binned, and fit for 10,000 repetitions to generate sampling distributions of model parameters. 

Uncertainty regions of the regression lines are the 95% confidence intervals generated from each bootstrapped fit over an interpolating region according to the variable’s domain, and they were displayed as shaded regions surrounding the fitted traces. Statistical significance of fitting parameters, where applicable, was determined from the BCa 95% confidence regions, which corresponds to a 5% α level (*p* = 0.05) [37]. Pairwise testing was performed on all pairs by a custom bootstrap algorithm of Welch’s T-test for unequal variances [39,40]. To account for multiple testing errors, all *p*-values were adjusted for false discovery rate [41].

## Data Availability

All of the data in the paper are available on request to the contributing author.
